# Illinois State Dental Society

**Published:** 1884-07

**Authors:** 


(TO BE CONTINUED.)

---

### ILLINOIS STATE DENTAL SOCIETY.

### TWENTIETH ANNUAL MEETING.

REPORTED EXPRESSLY FOR THE INDEPENDENT PRACTITIONER.

The twentieth annual meeting of the Illinois State Dental Society assembled in the Senate Chamber, at Springfield, Ill., Tuesday, May 13, 1884.

The meeting was called to order by the President, Dr. Edmund Noyes, of Chicago. Afterwards the roll was called by the Secretary, Dr. J. W. Wassall, of Chicago, showing an attendance of seventy-eight members, which was subsequently increased to ninety-eight.

Prayer was offered by the Rev. Dr. Post, of the Congregational Church.

Miscellaneous business came up at this juncture, and upon its completion the reports of committees and officers were heard. The election of new members was followed by the President's address—Vice-President H. H. Townsend, of Pontiac, in the chair. The address was a comprehensive review of the past year, with a prediction of a brighter future.

After the appointment of committees the society adjourned until 2 o'clock P. M.

AFTERNOON SESSION.

After the reading of the minutes of the morning session, and the transaction of other routine business, Dr. A. W. Harlan, of Chicago, from the committee to ascertain and report what arrangement, if any, had been made for dental representation at the proposed International Medical Congress at Copenhagen, on August 12–16th next, reported that after considerable inquiry, personal and by correspondence, he had been unable to learn that the plan of the proposed gathering contemplated a dental section; on the contrary, his assurances were in the negative. The report was agreed to.

A number of corresponding members from other States were present at this session, and several applicants for active membership being favorably reported upon, the candidates were elected by ballot.

The regular order was then taken up, and Dr. S. F. Duncan, of Wilmington, read a paper upon "Exposed Dental Pulps and Their Treatment."

The usual methods of capping and devitalization were discussed in this paper. Aconite was recommended for both local and internal use, and the stick of wood in preference to the barbed broach, for the removal of the pulp from root canals.

The oral discussion was opened by Dr. J. N. Crouse, of Chicago, who urged that to arrest the decay and prevent the destruction of the teeth, is the crowning work of operative dentistry.

He stated that a correct diagnosis of the actual condition of the pulp was of the first importance. If the pulp is exposed and living, the dentine at the point where the exposure will most likely be found, is usually sensitive. If the tooth is reasonably sensitive at the line of union of dentine and enamel, a condition of the pulp favorable to capping is indicated. He always leaves softened dentine over the point of exposure, and over the entire floor of the cavity, and caps over that. After excavating with care, he syringes the cavity forcibly, then dries it out and applies carbolic acid. This is allowed to remain several minutes. Is not particular to wipe out all the carbolic acid, sometimes leaving considerable. Children's teeth decay more rapidly than those of adults, and the pulps are relatively larger; consequently more caution is required in excavating. Has many times unexpectedly uncovered pulps in the sixth year molars. Aching teeth are treated for the relief of pain, and afterwards, at the same sitting, successfully capped and filled. This was not his former practice, but it is now, and he finds it justified by experience. He commenced the practice of capping exposed pulps with some doubt, he might say skepticism. The older practice was to remove the pulp, or at least a portion of it. But his later experience had fully demonstrated to his mind the utility of capping, and such was now his uniform practice, even in the case of children. Of course the condition of the pulp, whether abnormal or normal, was to be considered. If that was all right he proceeded to cap, and at once proceeded with the operation of filling. If the cap was carefully put on, there was little danger of subsequent aching of the tooth.

"I cap with whatever material seems best adapted to the case, often using oxy-chloride of zinc, and sometimes oxy-phosphate. I prefer the former, for the reason that it flows more easily over the opening. It does not cause pain, or but very little, when the pulp is previously treated with carbolic acid. Others differ with me regarding the frequency of capping. If those who object say it is not a good practice, I say to them, you have not applied the caps with sufficient skill (laughter), or you would succeed as I have. For fourteen years I have had good success in capping, and I can show cases I have examined three years and more after the operation, and found the pulps still alive."

*Dr. Spalding*—(of St. Louis). What proportion, relatively, does your success bear to your failures?

*Dr. Crouse*—(resuming). I estimate my failures, sir, at not more than one-tenth—perhaps less. If I have carefully done the work, success is pretty sure. I have had cases where I really supposed the pulps would die, but I capped nevertheless, and afterwards found them doing well. A boy called in my office only two days ago, for whom I had capped pulps five years ago. I examined them and found them alive and in good condition.

If filled temporarily, the cap must not be disturbed when the cavity is permanently filled. The state of the general health is not much considered. Have successfully capped pulps that have been exposed for years. Have capped many that I supposed would die, but they recovered.

*Dr. Patrick*—(of Belleville). I would inquire if, after thus treating it, you have removed the cap from the pulp, and with what result?

*Dr. Crouse*—I have often done this, and found the pulp alive and doing well. All aches and pains were gone. I have had patients come into my office in great pain, and after capping I rather expected them to come in the next day, perhaps howling with pain; but if they came, they did not complain at all. Why, sir! as skeptical a man as Dr. Dean, years after such capping, made an examination and found the pulp not destroyed.

I avoid wounding, but do not hesitate to cap a wounded pulp. Have tested many such cases and found the pulp alive years after capping. It is a very delicate operation, and unless skillfully done will not succeed.

*Dr. Newkirk*—How long have you used carbolic acid, and have you used creosote? If so, with what result—comparatively?

*Dr. Crouse*—I have not found creosote as reliable as carbolic acid. It does not generally produce so firm a coagulum. When it does, it may be that it was due to the impurity of the creosote, for much that was formerly used as creosote was, in fact, carbolic acid, or was largely mixed with it.

*Dr. Patrick.*—Is there any coagulum left, and if so, what becomes of it?

*Dr. Crouse.*—The coagulum is not disturbed.

Dr. Black subsequently explained that the coagulum is absorbed in the same manner that extravasated blood is.

*Dr. Patrick.*—Was sorry to say that in capping his success was not especially good, particularly when the pulps had been long exposed. Many years ago he was taught to trust to nature, and has followed the practice of removing the soft tissue and filling afterwards. He had been somewhat of an infidel in this matter, and was still skeptical. He had capped pulps with success, at times, but he took no risks.

*Dr. Sudduth.*—Said there was little trouble in capping pulps in ordinary cases, where the patient's condition justified it.

*Dr. Townsend.*—Said that his experience in capping was not wholly like that of Dr. Crouse.

The debate here became very animated and general, amid which Dr. Crouse was asked:

"What do you mean by saying that you expected patients to come in raving with pain as the result of the operation? Is it that you expected pain would follow the capping, and were surprised that it did not?"

*Dr. Crouse*—Yes, I expected that the patient might complain of pain. But I meant to be understood figuratively; not that I was surprised that such results did not always follow. Oh, no!

*Dr. Patrick.*—Ah! I thought the gentleman spoke of his general practice in preventing pain, not of an isolated case. (Laughter.)

*Dr. Crouse.*—Dr. Patrick has misconstrued my words. If he will come to Chicago I will show him the result of capping in frequent practice, and he will return to Belleville not an infidel, I am sure.

*Dr. Nelson.*—Inquired of Dr. Crouse whether his subsequent examination of pulps established the utility of capping.

*The President.*—Dr. Crouse has already said so.

*Dr. W. H. Eames.*—I came here from St. Louis in pursuit of information. You don't expect to learn much from me; but I would like to ask a few questions. I am much interested in this subject of capping pulps. Good operators have stood before us and explained their methods, but I would like to know somewhat further. First, I would like to ask Dr. Crouse how he decides upon the condition of the pulp—that it is healthy enough to justify capping? What evidence has he of this health, or what evidence does it require to convince him that it is proper to cap? Dr. Crouse says, if I understand him rightly, that the sensitiveness of the part is his criterion, and I ask him to tell at just what point to draw the line between the health of the pulp and its abnormal condition. I think that health has much to do with the justification, and with the result of the operation. Another matter would influence me in deciding upon the operation—that is age. The tooth-pulp of an adult might be capped, when that of a child ought not to be.

*Dr. Spalding.*—Being called upon, responded that more than twenty-five years ago, before the capping of pulps was practiced, he adopted the practice of devitalization and removal, and had not been sufficiently successful in capping pulps to induce him to change his general practice. Now, a few words relative to devitalization. About thirty-four years ago I ordered a druggist to fill the usual prescription employed for this purpose, viz., equal parts by weight of arsenious acid and of acetate morphia. Having in mind my knowledge of homeopathic pharmacy, I ordered the mass triturated half a day, for the purpose of being assured that the Ars. Ac. was minutely divided. I have used from the bottle containing this prescription ever since, and have distributed a part of its contents to others. Have met with none of the difficulties sometimes encountered in the use of arsenic, and I attribute my success to the finely divided condition of the active agent. Complaint is often made of difficulty and failure in destroying the vitality of the pulp near the extremity of the root canals. I do not hesitate to introduce this preparation into a root canal, and to convey it nearly or quite to the foramen at the apex. A mere dust on a dry broach is sufficient for this purpose. I have never observed any disagreeable consequences resulting from such practice.

*Dr. G. V. Black*—(of Jacksonville), said that he was already on record on this question, for he had expressed himself at former meetings. His earlier experiments in this pulp matter were not very successful. He would suggest that we differentiate, for cases vary in pathological condition. Thus, Dr. Crouse says if the pulp is normally sensitive, he decides to cap. I will only add that if I see an abnormal condition, I decide not to risk capping. I would hesitate more in a child's case than in that of an adult. The pulp is less and less important with age, and more important in childhood.

*Dr. Patrick.*—I am still inquisitive. Will Dr. Spalding be kind enough to tell me which element in the preparation acts first; the arsenious acid or the morphia? I came here, two or three hundred miles, for information.

*Dr. Spalding*—Would cheerfully explain. He used morphia for the simple reason that others had done so before him. Thought it probable that some other substance, say some neutral substance, would answer just as good a purpose as the morphia. It was the arsenious acid that did the work, and so far as he knew, the morphia only served to increase the bulk, and thus lessen the amount of Ars. Ac. applied.

